# Hydrogen-Bond Dissociation Energies from the Properties of Isolated Monomers

**DOI:** 10.1021/acs.jpca.3c02159

**Published:** 2023-05-19

**Authors:** Ibon Alkorta, Anthony Legon

**Affiliations:** † Instituto de Química Médica (IQM-CSIC), Juan de la Cierva, 3, E-28006 Madrid, Spain; ‡ School of Chemistry, University of Bristol, Cantock’s Close, Bristol BS8 1TS, U.K.

## Abstract

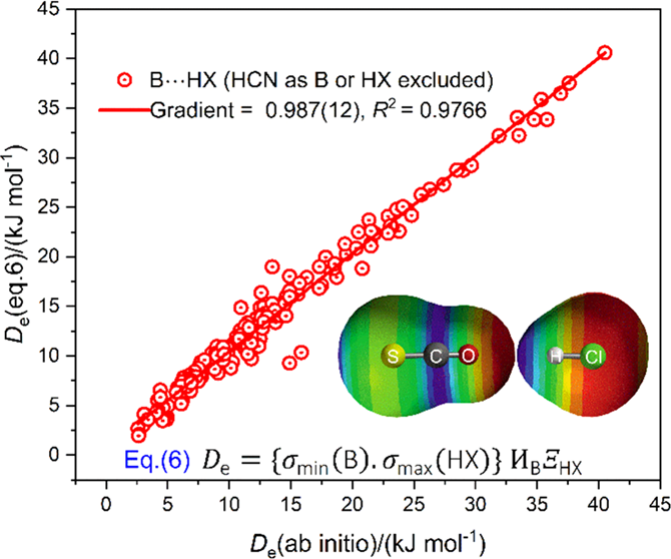

The strength of binding, as measured by the equilibrium dissociation energy *D*_e_ of an isolated hydrogen-bonded complex B···HX, where B is a simple Lewis base and X = F, Cl, Br, I, CN, CCH, or CP, can be determined from the properties of the infinitely separated components B and HX. The properties in question are the maximum and minimum values σ_max_(HX) and σ_min_(B) of the molecular electrostatic surface potentials on the 0.001 e/bohr^3^ iso-surfaces of HX and B, respectively, and two recently defined quantities: the reduced electrophilicity Ξ_HX_ of HX and the reduced nucleophilicity И_B_ of B. It is shown that *D*_e_ is given by the expression *D*_e_ = {σ_max_(HX)σ_min_(B)} И_B_ Ξ_HX_. This is tested by comparing *D*_e_ calculated ab initio at the CCSD(T)(F12c)/cc-pVDZ-F12 level of theory with that obtained from the equation. A large number of complexes (203) falling into four categories involving different types of hydrogen-bonded complex B···HX are investigated: those in which the hydrogen-bond acceptor atom of B is either oxygen or nitrogen, or carbon or boron. The comparison reveals that the proposed equation leads to *D*_e_ values in good agreement in general with those calculated ab initio.

## 1 Introduction

It has long been an aim of those concerned with noncovalent interactions of molecules, particularly the hydrogen bond, to predict the properties of the complex so formed from those of the separate components. For example, Drago and co-workers proposed a relationship between the dissociation enthalpy of hydrogen-bonded complexes and two parameters associated with the separate components, one assigned to the hydrogen-bond donor and the other assigned to the hydrogen-bond acceptor.^1,2^ The approach favored by Taft and Abraham utilized experimental acidity and basicity scales of the hydrogen-bond donor and acceptor molecules, respectively,^3−7^ while Platts employed acidities and basicities calculated theoretically.^8−11^ Alternative approaches^12−14^ discussed hydrogen bonding in complexes B···HX in terms of the distances *r*(B···H) and *r*(HX) and also via the relationship between electron density properties and *r*(B···H) distances.^15−20^ From geometries determined in microwave spectroscopic studies of various hydrogen-bonded complexes B···HX (X is a halogen atom), rules for predicting angular geometries based on HX acting as a probe for the directions of nonbonding and π-bonding electron pairs were formulated and discussed in several articles.^21−23^ The rules were electrostatic in character. Buckingham and Fowler^24,25^ were able to predict successfully the angular geometries of hydrogen-bonded complexes in terms of the electric-charge distribution of the separate molecules, each described by a distributed multipole analysis. In the present article, we propose a method of predicting two measures of the strength of the hydrogen bond, namely, the equilibrium dissociation energy *D*_e_ for the process B···HX = B + HX and the intermolecular quadratic, stretching force constant *k*_σ_ of the complex B···HX from the properties of the isolated molecules B and HX. The properties in question are the so-called molecular electrostatic surface potentials (MESP) of B and HX together with the reduced nucleophilicity of the acceptor molecule B and the reduced electrophilicity of the hydrogen-bond donor HX, as recently introduced.

## 2 Theoretical Methods

The equilibrium dissociation energies *D*_e_ of most of the hydrogen-bonded complexes used to produce the generalizations presented here are taken from recent publications.^26−28^ For complexes not considered in earlier publications, their geometries and those of the isolated monomers were calculated by exactly same the approach as in refs (26−28). Thus, geometry optimizations were conducted at the CCSD(T)(F12c) computational level^29,30^ in the frozen core approximation and with the choice of cc-pVDZ-F12 basis sets,^31^ using the MOLPRO program.^32^*D*_e_ values were taken as the difference of the electronic energies of the complex and those of the isolated monomers and, as previously, were corrected for basis set superposition error (BSSE) by means of the full counterpoise method of Boys and Bernadi.^33^ The molecular electrostatic surface potentials (MESP) of the isolated Lewis bases B and acids HX were calculated with the GAUSSIAN program^34^ by employing the MP2/aug-cc-pVTZ wavefunction and analyzed on the 0.001 e/bohr^3^ electron density iso-surface with the Multiwfn program.^35^

## 3 Results

### 3.1 Background

It was shown as long ago as 1987^36^ that the intermolecular stretching force constant *k*_σ_ for an isolated hydrogen-bonded complex B···HX (available from the spectroscopically determined centrifugal distortion constant)^37^ can be predicted from the expression
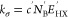
1in which *N*_B_^′^ and *E*_HX_^′^ are the nucleophilicities and electrophilicities of the Lewis base B and the Lewis acid HX, respectively, and *c*′ is a constant conveniently chosen as 1.0 N m^–1^ so that *N*_B_^′^ and *E*_HX_^′^ are dimensionless. Later, it was shown^38^ that *k*_σ_ is directly proportional to the energy, *D*_e_ , required to dissociate B···HX from its equilibrium conformation to the infinitely separated B and HX molecules and therefore that eq 1 can be written as
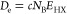
2where *c* is a constant conveniently defined as 1 kJ mol^–1^ so that if *D*_e_ has units of kJ mol^–1^, *N*_B_ and *E*_HX_ are dimensionless. Equation 2 has been extensively tested for a wide range of complexes involving many Lewis bases B in numerous complexes with several types of Lewis acid A [hydrogen-bonded B···HX,^39−42^ halogen-bonded B···XY (X,Y are halogen atoms),^39−42^ coinage metal complexes B···MX (M = Cu, Ag, Au),^43^ alkali-metal complexes B···MX (M = Li, Na, K),^44^ and alkaline-earth metal complexes B···MR_2_ (M = Be, Mg; R = H, F, CH_3_)^45^]. Thereby, the nucleophilicity *N*_B_ of many Lewis bases B and the electrophilicity *E*_A_ of many Lewis acids A in isolation have been established. Equation 2 was verified by establishing that graphs of *D*_e_ versus *E*_A_ were indeed straight lines through the origin, within the errors of the linear regression fits when B was held constant and A was varied, and likewise for graphs of *D*_e_ vs *N*_B_ when A was held constant and B was varied.

**Figure 1:**
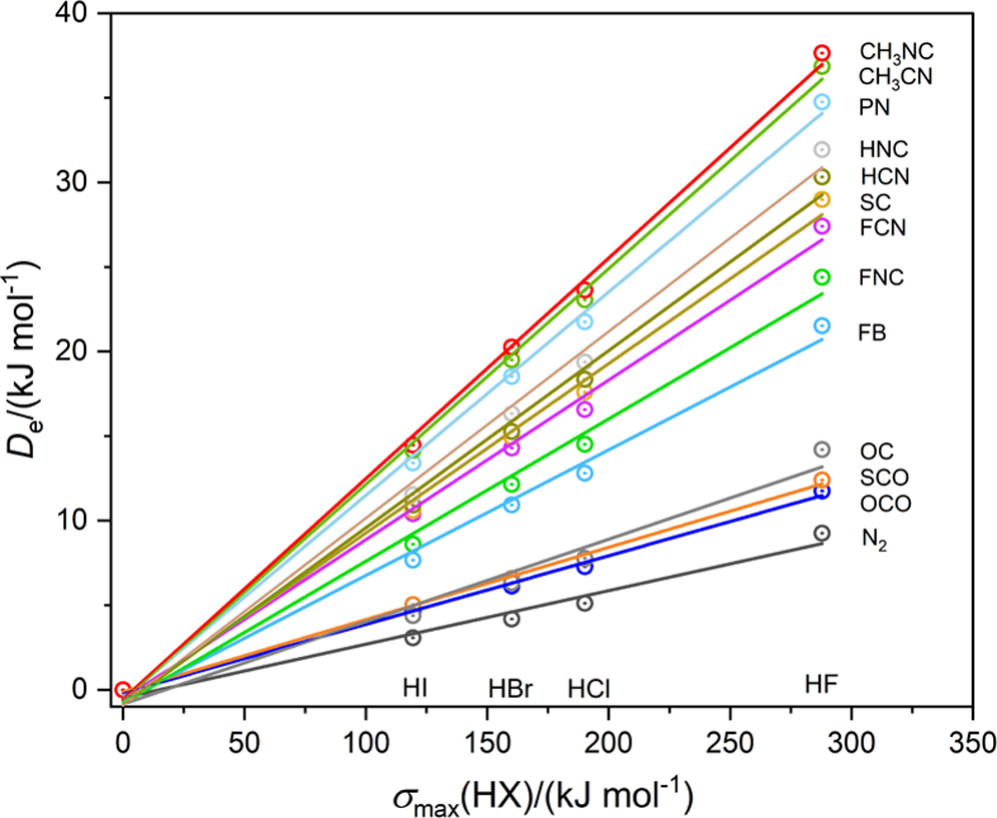
Graphs of *D*_e_ versus σ_max_(HX) for complexes B···HX. Lewis bases B are labeled in the right-hand column, while Lewis acids are indicated in the row along the abscissa. Data from refs (26) and (27).

**Figure 2:**
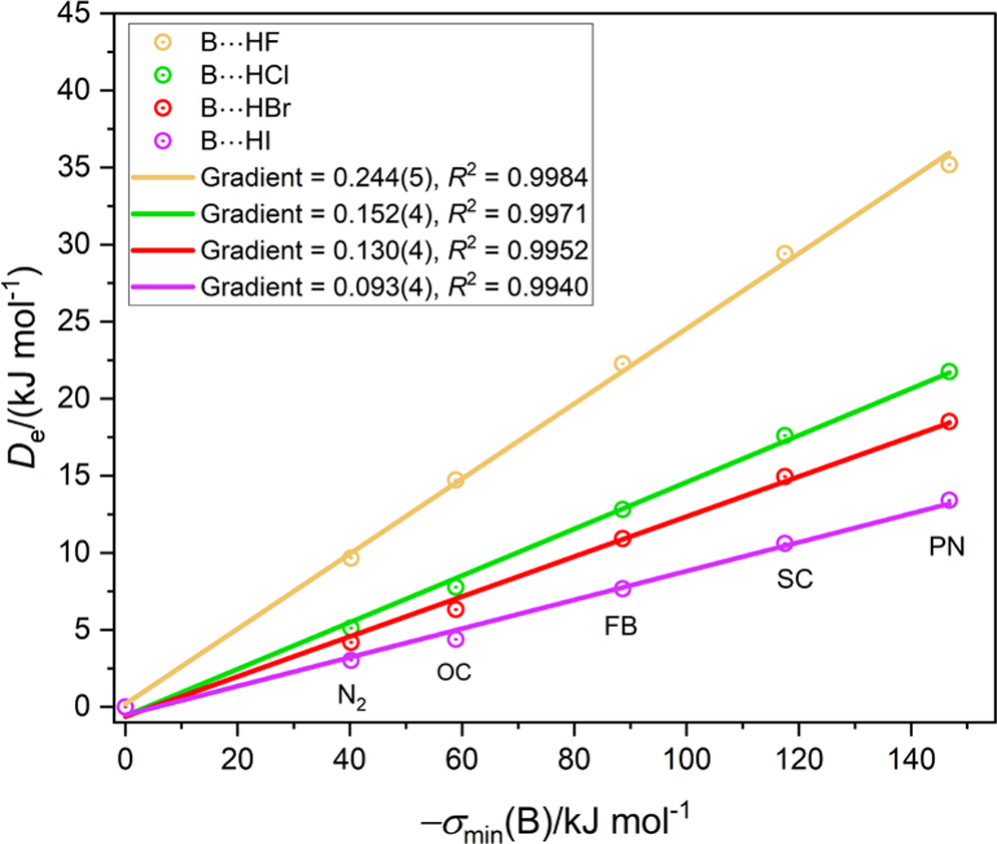
Graphs of *D*_e_ vs −σ_min_(B) for complexes B···HX. The five Lewis bases B are indicated. Points for the Lewis acids HF, HCl, HBr, and HI are identified in the inset. Data are from refs (27) and (28).

An important molecular property in the present context is the quantity called the molecular electrostatic surface potential (MESP).^46^ This property of a molecule is defined as the electrostatic potential energy of a unit positive charge on the iso-surface at which the electron density has a constant value, in this case 0.001 e/bohr^3^. Given that noncovalent interactions have a substantial electrostatic component, that the axes of nonbonding electron pairs or π-bonding pairs are directions associated with most negative (minimum) electrostatic potential, and that the atoms acting as donors in hydrogen bond, halogen bond, etc. formation are associated with regions of maximum electrostatic potential, it seems reasonable that the MESP has a role when noncovalent interactions are discussed. This is made clear by Figure 1, which shows plots of *D*_e_ for 13 different series of complexes B···HX versus the maximum value σ_max_(HX), the MESP associated with HX (X = F, Cl, Br, and I), along the abscissa. The Lewis bases are B = CH_3_NC, CH_3_CN, PN, HNC, HCN, SC, FCN, FNC, FB, OC, SCO, OCO, and N_2_ (as labeled in the vertical column on the right-hand side). The MESPs were calculated at the MP2/aug-cc-pVTZ level on the 0.001 e/bohr^3^ iso-surface for each HX. The σ_max_(HX) values lie on the molecular axis near to the H atom. The points in Figure 1 were fitted by linear regression to yield the continuous solid lines, one for each of the 13 series B···HX. The quality of each fit is excellent, given that the parameters *R*^2^ of the regression fits have values greater than 0.99 for all series, except those involving B = N_2_ and CO, for which the values are 0.978 and 0.973, respectively. The corresponding graph of *D*_e_ versus −σ_min_(B) is shown in Figure 2 for five diatomic Lewis bases. Again the linear regression fits are excellent, with all *R*^2^ greater than 0.994. The data for Figures 1 and 2 are available in refs (26−28). Figures 1 and 2 indicate that there exists an intimate connection between *D*_e_ and the maximum and minimum values of the MESPs of B and HX, respectively, and this suggested the following procedure. Division of eq 2 (with *c* = 1 kJ mol^–1^ now implied) by σ_max_(HX) gives
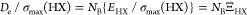
3while division of eq 2 by σ_min_(B) results in
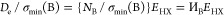
4When *D*_e_/σ_max_(HX) was plotted against *N*_B_ for complexes B···HX (X = F, Cl, Br, I), the separate straight lines through the origin (generally having different gradients) that were previously obtained from the *D*_e_ versus *N*_B_ plots for different molecules B became conflated to a single straight line. This led to the definition of the quantity Ξ_HX_ = *E*_HX_/σ_max_(HX) as the reduced electrophilicity, a property common to the HX molecules, independent of whether F, Cl, Br, or I was attached to H. Likewise, when *D*_e_/σ_min_(B) was plotted against *E*_HX_ for a range of Lewis bases B, the separate straight lines through the origin obtained from the *D*_e_ vs *E*_HX_ plots (generally of different gradients) became conflated to a single straight line and suggested the definition of И_B_ = *N*_B_/σ_min_(B) as the reduced nucleophilicity of the group of Lewis bases B involved. Analysis in ref (28) showed that values of И_B_ determined from different types of Lewis base were not significantly different when the atom of B directly involved in the hydrogen bond in B···HX was the same and hence that И_B_ is an intrinsic property of the atom, independent of the remainder of B. Moreover, И_B_ vary little when that atom was any one of the first-row series B, C, N, or O.

The starting point of the analysis presented in this article is to note that if eq 3 is divided by σ_min_(B) or eq 4 is divided by σ_max_(HX), the result is
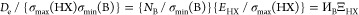
5which rearranges to
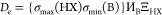
6We note that the quantities on the right-hand side of eq 6 are properties of the isolated Lewis base B and the isolated Lewis acid HX. Hence, eq 6 suggests a route to the calculation of the dissociation energy of an isolated hydrogen-bonded complex B···HX from the properties of the individual molecules B and HX.

### 3.2 Comparison of *D*_e_ Calculated from Eq 6 and Ab Initio Calculated Values

Table 1 records values of σ_max_(HX) and σ_min_(B), as calculated at the MP2/aug-cc-pVTZ level, for the molecules B and HX to be considered here. Table 2 carries values of the reduced electrophilicities Ξ_HX_ and the reduced nucleophilicities И_B_ of the molecules of interest, as determined in refs (26) and (28), respectively. Groups of complexes B···HX in which the hydrogen bond to HX is to O are discussed first, followed by corresponding groups in which the bond is successively to N, C, and B atoms.

**Table 1:** Minimum σ_min_(B) and Maximum σ_max_(HX) Values of Molecular Electrostatic Surface Potentials for Lewis Bases and Lewis Acids, Respectively, Calculated at the MP2/aug-cc-pVTZ Level

lewis base B	σ_min_(B)/(kJ mol^–1^)	lewis base B	σ_min_(B)/(kJ mol^–1^)	lewis acid HX	σ_max_(HX)/(kJ mol^–1^)
H_3_C–B	–160.3	O≡C	–58.5	HF	289.7
H_3_Si–B	–133.7	S≡C	–119.7	HCl	190.2
H–B	–134.5	CH_3_CN	–159.2	HBr	160.1
F–B	–89.3	HC≡N	–133.7	HI	119.4
Cl–B	–103.7	FC≡N	–119.1	HC≡N	216.9
Br–B	–99.0	N≡N	–35.8	HC≡CH	134.8
I–B	–90.9	P≡N	–101.5	HC≡P	126.3
N≡C–B	–91.4	O=C=O	–44.6		
C≡N–B	–95.5	S=C=O	–46.2		
F_3_C–B	–83.3	C≡O	–25.0		
CH_3_N≡C	–161.8	H_2_O	–135.1		
HN≡C	–138.9	H_2_C=O	–121.4		
FN≡C	–106.9	H_2_C=C=O	–64.5		

**Table 2:** Values of the Reduced Nucleophilicity И_B_ and Reduced Electrophilicity Ξ_HX_ of Lewis Bases B and Acids HX

type of complex	H-bond acceptor atom of base B	И_B_^a^	type of HX molecule	Ξ_HX_^b^
R–boron···HX^c^	boron	0.0368	X = F, Cl, Br, I	0.0239
			X = CN, CCH, CP	0.0164
R–N≡C···HX^c^	carbon	0.0337		
O≡C···HX	carbon	0.0349		
S≡C···HX	carbon	0.0349		
R–C≡N···HX^c^	nitrogen	0.0333		
N≡N···HX	nitrogen	0.0374		
P≡N···HX	nitrogen	0.0374		
C≡O···HX	oxygen	0.0376		
O=C=O···HX	oxygen	0.0376		
S=C=O···HX	oxygen	0.0376		
H_2_O···HX	oxygen	0.0386		
H_2_C=O···HX	oxygen	0.0386		
H_2_C=C=O···HX	oxygen	0.0386		

a From ref (28).

b From ref (26).

c R = H_3_C, H, F.

#### 3.2.1 Complexes B···HX in Which the Hydrogen-Bond Acceptor Atom Is O

**Figure 3:**
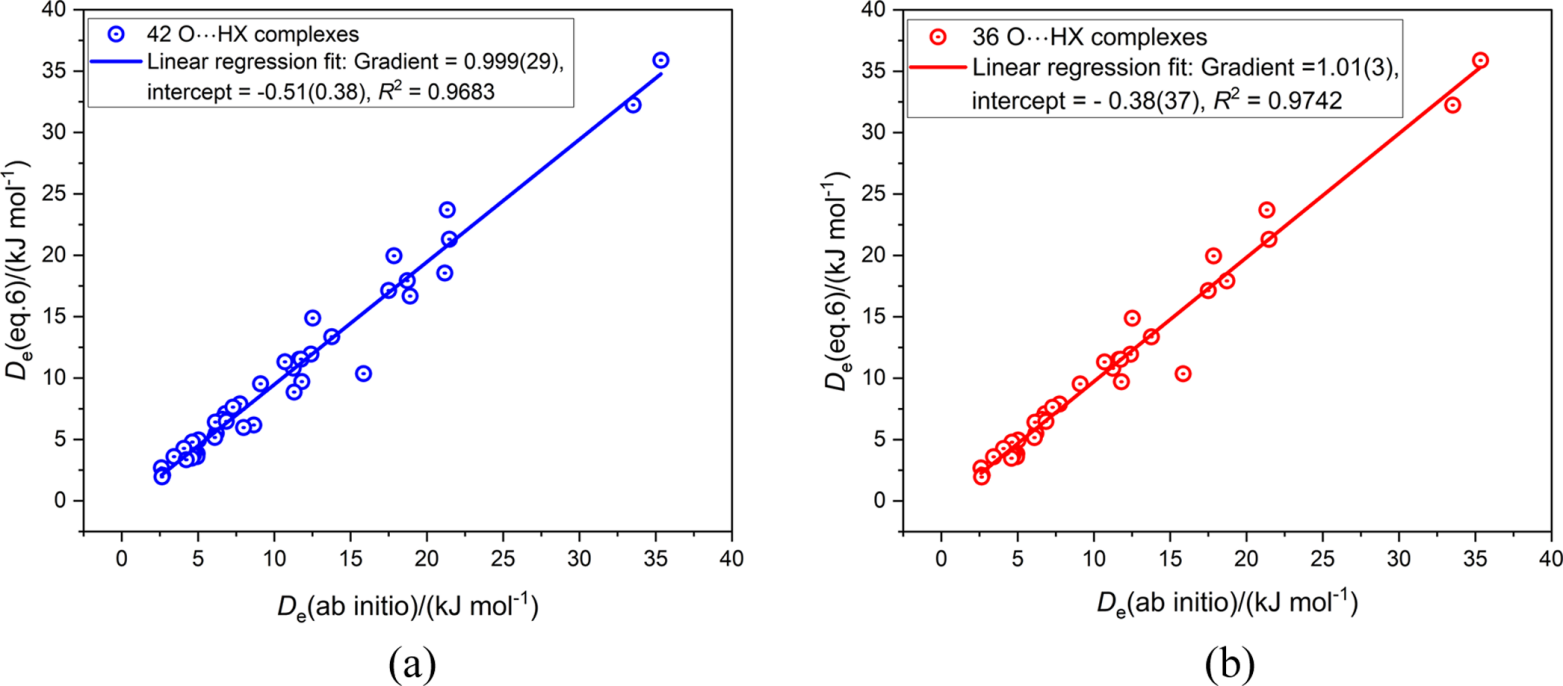
(a) Comparison of *D*_e_ of B···HX calculated from eq 6 with those obtained by ab initio calculations at the CCSD(T)(F12c)/cc-pVDZ-F12 level for 42 complexes containing the O···HX hydrogen bond. (b) Same comparison after complexes containing HCN were removed from the plot.

Table 3 lists the complexes containing the O···HX hydrogen bond (X =F, Cl, Br, I, CN, CCH, CP). The Lewis bases involved are H_2_O, H_2_C=O, H_2_C=C=O, S=C=O, O=C=O and C≡O. In columns 2 and 3 are σ_min_(B)/(kJ mol^–1^) and σ_max_(HX)/(kJ mol^–1^), respectively. Columns 4 and 5 carry values of the reduced nucleophilicities and electrophilicities И_B_ and Ξ_HX_, respectively (from Table 2), while the final three columns show *D*_e_ (eq 6), *D*_e_ (ab initio), and the difference between these two values, respectively. The agreement between *D*_e_ (eq 6) and *D*_e_ (ab initio) is mainly good, except perhaps for the complexes B···HCN. In fact, it will be noted later that HCN is generally anomalous in this respect, for reasons presently unknown. An interesting feature of Table 3 is the group of complexes CO···HX, in which the hydrogen bond is to the oxygen atom of carbon monoxide. In fact, both ends of carbon monoxide are negative and capable of sustaining hydrogen bonds, that is CO is ambi-nucleophilic, as may be seen from the two values of σ_min_(CO) included in Table 1. This is also true of N_2_. It will be seen in Section 3.2.2 that the other isomer, OC···HX, is the more strongly bound. It is worth noting that, although the molecules B involved in Table 3 range from diatomics, through linear triatomics, to polyatomic asymmetric tops, the predictions of *D*_e_ by eq 6 are all good, except for those involving O···HCN, as mentioned earlier. Figure 3a shows a graph of *D*_e_ (eq 6) plotted against *D*_e_ (ab initio) for the 42 O···HX complexes listed in Table 3. The continuous straight line is the result of a linear regression fit to all of the points and has a gradient of 0.999(29) and *R*^2^ = 0.9683. The fit is slightly better if the O···HCN points are excluded, as can be seen in Figure 3b.

**Table 3:** Comparison of *D*_e_ (Ab Initio) and *D*_e_ (Eq 6) for Complexes with O⋯HX Hydrogen Bonds

B···HX	σ_min_(B)/(kJ mol^–1^)	σ_max_(HX)/(kJ mol^–1^)	И_B_ from Table 2	Ξ_HX_ from Table 2	*D*_e_ (eq 6)/(kJ mol^–1^)	*D*_e_ (ab initio)/(kJ mol^–1^)	diff. in *D*_e_/(kJ mol^–1^)
H_2_O···HF	–135.1	287.9	0.0386	0.0239	35.9	35.3	–0.6
H_2_O···HCl	–135.1	190.2	0.0386	0.0239	23.7	21.3	–2.4
H_2_O···HBr	–135.1	160.1	0.0386	0.0239	20.0	17.8	–2.2
H_2_O···HI	–135.1	119.4	0.0386	0.0239	14.9	12.5	–2.3
H_2_O···HCN	–135.1	216.9	0.0386	0.0164	18.6	21.2	2.6
H_2_O···HCCH	–135.1	134.8	0.0386	0.0164	11.5	11.6	0.1
H_2_O···HCP	–135.1	126.3	0.0386	0.0164	10.8	11.2	0.4
H_2_CO···HF	–121.4	287.9	0.0386	0.0239	32.2	33.5	1.3
H_2_CO···HCl	–121.4	190.2	0.0386	0.0239	21.3	21.5	0.2
H_2_CO···HBr	–121.4	160.1	0.0386	0.0239	17.9	18.7	0.8
H_2_CO···HI	–121.4	119.4	0.0386	0.0239	13.4	13.8	0.4
H_2_CO···HCN	–121.4	216.9	0.0386	0.0164	16.7	18.9	2.2
H_2_CO···HCCH	–121.4	134.8	0.0386	0.0164	10.4	15.9	5.5
H_2_CO···HCP	–121.4	126.3	0.0386	0.0164	9.7	11.8	2.1
H_2_CCO···HF	–64.5	287.9	0.0386	0.0239	17.1	17.5	0.4
H_2_CCO···HCl	–64.5	190.2	0.0386	0.0239	11.3	10.7	–0.6
H_2_CCO···HBr	–64.5	160.1	0.0386	0.0239	9.5	9.1	–0.4
H_2_CCO···HI	–64.5	119.4	0.0386	0.0239	7.1	6.8	–0.3
H_2_CCO···HCN	–64.5	216.9	0.0386	0.0164	8.9	11.3	2.4
H_2_CCO···HCCH	–64.5	134.8	0.0386	0.0164	5.5	6.2	0.7
H_2_CCO···HCP	–64.5	126.3	0.0386	0.0164	5.2	6.1	0.9
SCO···HF	–42.6	287.9	0.0376	0.0239	12.0	12.4	0.4
SCO···HCl	–46.2	190.2	0.0376	0.0239	7.9	7.7	–0.2
SCO···HBr	–46.2	160.1	0.0376	0.0239	6.6	6.6	–0.1
SCO···HI	–46.2	119.4	0.0376	0.0239	5.0	5.0	0.0
SCO···HCN	–46.2	216.9	0.0376	0.0164	6.2	8.7	2.5
SCO···HCCH	–46.2	134.8	0.0376	0.0164	3.8	4.9	1.1
SCO···HCP	–46.2	126.3	0.0376	0.0164	3.6	4.9	1.3
OCO···HF	–44.6	287.9	0.0376	0.0239	11.5	11.8	0.2
OCO···HCl	–44.6	190.2	0.0376	0.0239	7.6	7.3	–0.3
OCO···HBr	–44.6	160.1	0.0376	0.0239	6.4	6.1	–0.3
OCO···HI	–44.6	119.4	0.0376	0.0239	4.8	4.6	–0.2
OCO···HCN	–44.6	216.9	0.0376	0.0164	6.0	8.0	2.0
OCO···HCCH	–44.6	134.8	0.0376	0.0164	3.7	4.7	1.0
OCO···HCP	–44.6	126.3	0.0376	0.0164	3.5	4.6	1.1
CO···HF	–25.0	287.9	0.0376	0.0239	6.5	6.9	0.4
CO···HCl	–25.0	190.2	0.0376	0.0239	4.3	4.1	–0.2
CO···HBr	–25.0	160.1	0.0376	0.0239	3.6	3.4	–0.2
CO···HI	–25.0	119.4	0.0376	0.0239	2.7	2.6	–0.1
CO···HCN	–25.0	216.9	0.0376	0.0164	3.3	4.2	0.9
CO···HCCH	–25.0	134.8	0.0376	0.0164	2.1	2.7	0.6
CO···HCP	–25.0	126.3	0.0376	0.0164	1.9	2.6	0.7

#### 3.2.2 Complexes B···HX in Which the Hydrogen-Bond Acceptor Atom of Base B Is Nitrogen

**Figure 4:**
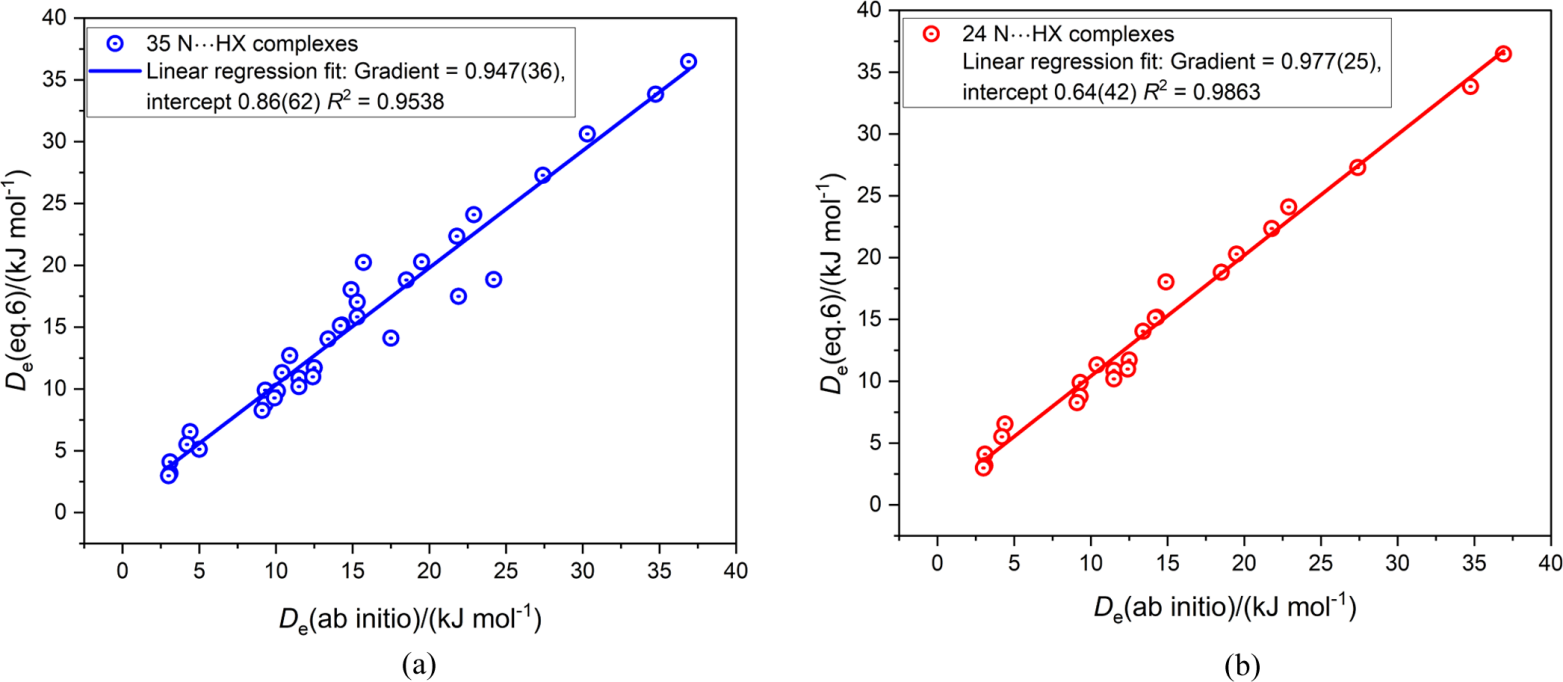
(a) Comparison of *D*_e_ of B···HX calculated from eq 6 with those obtained by ab initio calculations for 35 complexes containing N···HX hydrogen bonds. (b) Same comparison when the 11 complexes having HCN as either the hydrogen-bond donor or acceptor are removed.

Values of the equilibrium dissociation energy *D*_e_ for 35 complexes containing the N···HX hydrogen bond (X = F, Cl, Br, I, CN, CCH, CP), as calculated ab initio and via eq 6 are listed in Table S1, which is available in the Supporting Information. The graph of all *D*_e_ (eq 6) plotted against *D*_e_ (ab initio) is shown in Figure 4a. The complexes consist of CH_3_CN···HX, HCN···HX, FCN···HX, N_2_···HX, and PN···HX. Complexes involving NH_3_ and several amines have been excluded for reasons explained in ref (26). There is considerable scatter of the points in Figure 4a. When the points involving HCN either as proton donor or acceptor are removed, the scatter is reduced with the gradient and *R*^2^ of the regression fit both closer to 1, as can be seen in Figure 4b.

#### 3.2.3 Complexes B···HX in Which the Hydrogen-Bond Acceptor Atom of the Base B Is Carbon

**Figure 5:**
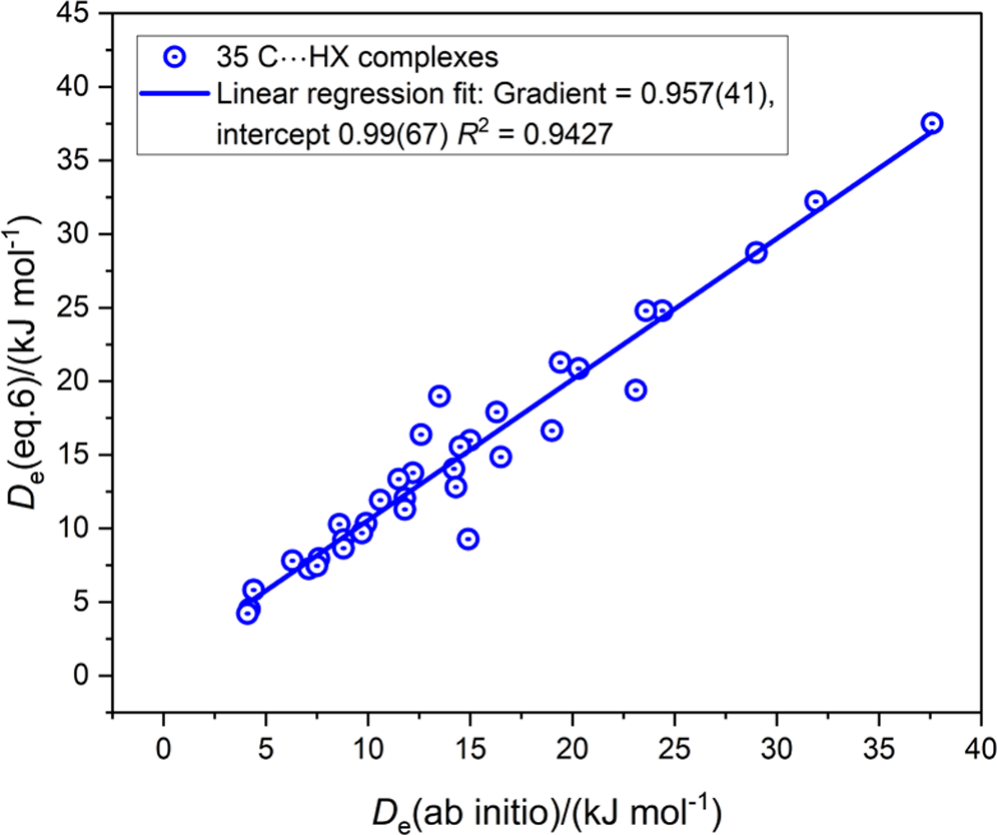
Comparison of *D*_e_ of B···HX calculated from eq 6 with those obtained by ab initio calculations for 35 complexes containing C···HX hydrogen bonds.

Values of the equilibrium dissociation energy *D*_e_ for 35 complexes containing the C···HX hydrogen bond (X = F, Cl, Br, I, CN, CCH, CP) calculated ab initio and via eq 6 are listed in Table S2, which is available in the Supporting Information. The graph of all *D*_e_ (eq 6) plotted against *D*_e_ (ab initio) is shown in Figure 5. The complexes consist of CH_3_NC···HX, HNC···HX, FNC···HX, OC···HX, and SC···HX. Guided by the anomalous behavior of HCN complexes when the H-bond acceptor atom is O or N, the five hydrogen bonds of the type C···HCN type (with HCN as H-bond donor) were removed from Figure 5, with the result shown in Figure S1. Again there is a small improvement in the scatter of points, as indicated by the movement of both the gradient and *R*^2^ both closer to 1.0 than in Figure 5.

#### 3.2.4 Complexes B···HX in Which the Hydrogen-Bond Acceptor Atom of the Lewis Base Is Boron

**Figure 6:**
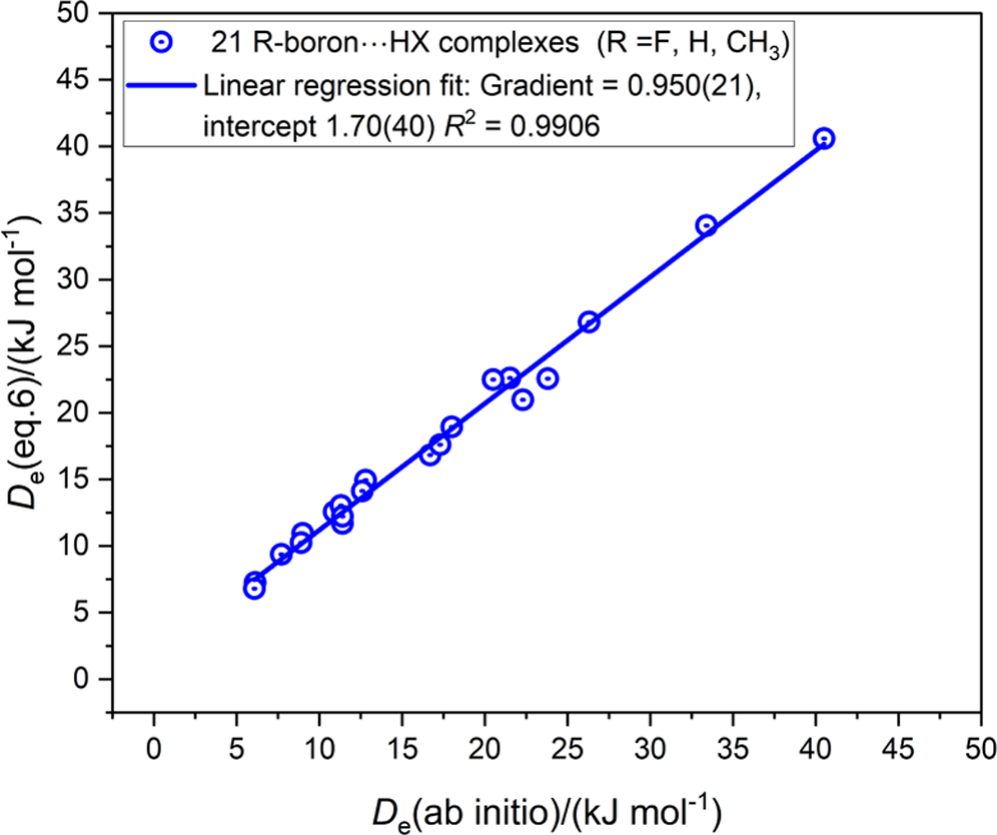
Comparison of *D*_e_ of B···HX calculated from eq 6 with those obtained by ab initio calculations for 21 complexes containing boron···HX hydrogen bonds.

The compound F–B has been characterized experimentally,^47^ and theory has shown^48^ that the predominant contribution to its valence-bond description is a Lewis structure having a single covalent bond, three equivalent nonbonding electron pairs on F, and one nonbonding pair on the axis of FB. Hence, F–B should form hydrogen bonds with the Lewis acids HX (X =F, Cl, Br, I, HCN, HCCH, and HCP) of the type F–B···HX. Complexes R–B···HX in which R is a monovalent group such as F, H, and CH_3_ have been characterized by ab initio calculations, and their equilibrium dissociation energies obtained are recorded in ref (27). Moreover, several R–B···HX complexes were used as the basis for a quantitative measure of the inductive effect of various groups R in isolated R–B molecules.^27^ The values of *D*_e_ for the 21 hydrogen-bonded complexes R–B···HX (R =F, H, and CH_3_; X = F, Cl, Br, I, CN, CCH, and CP) calculated at the CCSDT(F12c)/cc-pVDZ-F12 level are available in ref (27) and were used there to deduce the value И_RB_ = 0.0368(10) for the reduced nucleophilicity appropriate to R = F, H, and CH_3_. This quantity is used in eq 6 to obtain the values of *D*_e_ (eq 6) for the 21 R–B···HX complexes that are recorded in Table S3 in the Supporting Information, along with their ab initio calculated counterparts *D*_e_ (ab initio) and the differences between these two quantities. Figure 6 shows the graph of *D*_e_ (eq 6) as the ordinate and *D*_e_ (ab initio) as the abscissa for these complexes. The corresponding plot when the three complexes F–B···HCN, H–B···HCN, and H_3_C–B···HCN are removed is shown in Figure S2 in the Supporting Information. The fit quality is only slightly improved from that in Figure 6.

### 3.3 Predictions of *D*_e_ with Eq 6 for Complexes B···HX That Were Not Used in Generating И_B_ and Ξ_HX_

**Figure 7:**
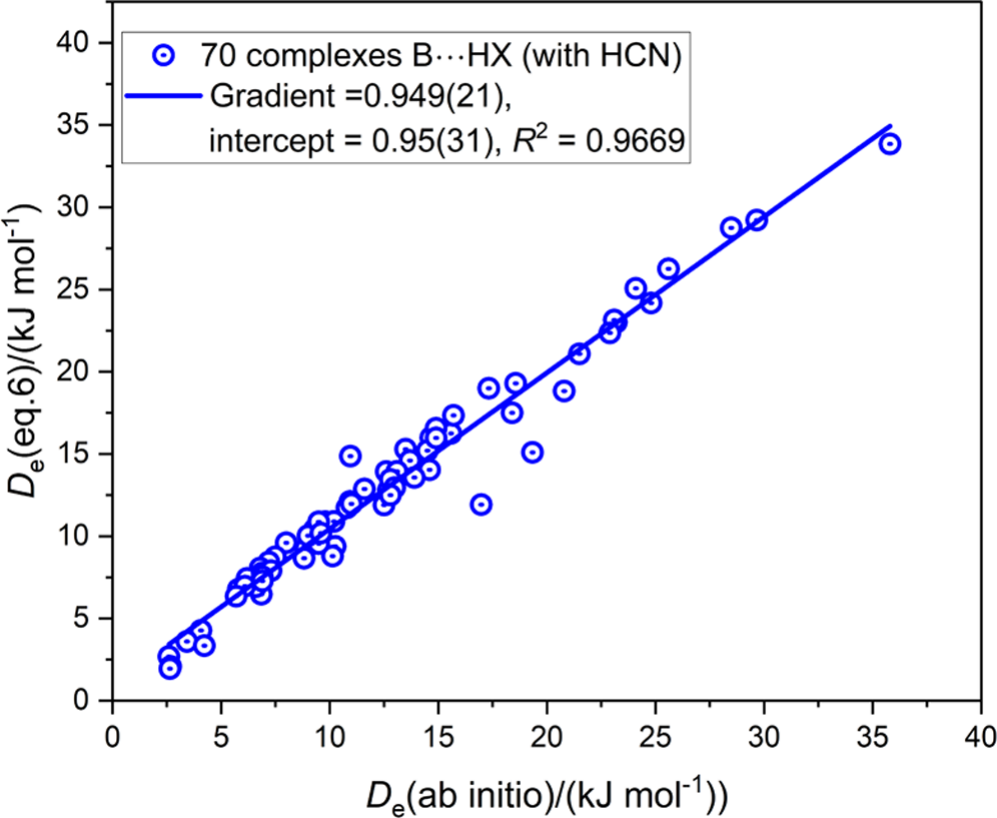
Comparison of *D*_e_ for 70 complexes B···HX calculated from eq 6 with those obtained by ab initio calculations. The complexes involve the Lewis bases CO, ClCN, ClNC, and R–B (with R = H_3_Si, Cl, Br, I, CN, NC, and F_3_C) and were chosen because they were not involved in the determination of the reduced nucleophilicities И_B_ or the reduced electrophilicities Ξ_HX_.

Figures 3–6 and Tables 1 and S1–S3 are encouraging in that they show that the equilibrium dissociation energy *D*_e_ of simple complexes of the type B···HX (X = F. Cl, Br, I, CN, CCH, and CP) can be predicted with reasonable accuracy from the properties of the isolated molecules B and HX, namely, σ_min_(B) and σ_max_(HX), the reduced nucleophilicity И_B_ of B, and the reduced electrophilicity Ξ_HX_ of HX. However, И_B_ and Ξ_HX_ were determined by use of the *D*_e_ values of all of the complexes listed in Tables 1 and S1 (except the values for CO···HX), S2, and S3. It remains to apply a more stringent test, that is to calculate *D*_e_ from eq 6 and compare those with a set calculated ab initio for complexes B···HX that were not used in generating И_B_ and Ξ_HX_. The *D*_e_ (eq 6) and *D*_e_ (ab initio) values for the CO···HX complexes are in Table 1, while Table S4 carries these quantities for the complexes, ClCN···HX, ClNC···HX, and R–B···HX, where R = H_3_Si, Cl, Br, I, CN, NC, and F_3_C. None of these were used to determine И_B_ and Ξ_HX_. The graph of *D*_e_ (eq 6) vs *D*_e_ (ab initio) is displayed in Figure 7 for the full set of these 70 complexes. The resulting points show some scatter with respect to the regression fit, with *R*^2^ = 0.9669 and a gradient = 0.949(21). When the 10 complexes that employ HCN as the Lewis acid are removed, the scatter is significantly reduced, with the result shown in Figure S3 in the Supporting Information. Thus, the value of *R*^2^ and the gradient increase to 0.9833, and 0.963(17), respectively. It is concluded from these observations that, for hydrogen-boned complexes formed between simple molecules of the type considered here, eq 6 can predict with reasonable accuracy the value of *D*_e_, even when these complexes were not involved in the determination of the reduced quantities И_B_ and Ξ_HX_. Moreover, the range of *D*_e_ values that can be predicted from these properties of the separate molecules is significant, from about 4 to 40 kJ mol^–1^.

### 3.4 Calculation of Intermolecular Stretching Force Constants *k*_σ_ from *D*_e_

It was shown in ref (38) that, for a wide range of hydrogen-bonded and halogen-bonded complexes, the quadratic intermolecular stretching force constant *k*_σ_ is directly proportional to the dissociation energy *D*_e_. Experimental values of *k*_σ_ can be determined from centrifugal distortion constants available from analysis of rotational spectra using the expressions due to Millen^37^ or can be calculated ab initio by finding the second derivative of the potential energy with respect to the intermolecular distance evaluated at the equilibrium.^49^ The former values have necessarily employed zero-point spectroscopic constants in the Millen formulae, so the latter are preferred here. It was shown in ref (49) that when ab initio values of *D*_e_ calculated at the CCSD(T)/CBS level were plotted against *k*_σ_ for hydrogen-bonded B···HX of type considered here, the appropriate form of eq 1 combined with eq 2 for complexes of the type considered here is
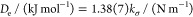
7Equation 7 therefore provides a means of predicting *k*_σ_ from the properties of the isolated molecules B and HX.

## 4 Conclusions

The equilibrium dissociation energies *D*_e_ of 203 hydrogen-bonded complexes of the type B···HX, in which B is a simple Lewis base molecule and X is one of F, Cl, Br, I, CN, CCH, or CP, have been predicted by means of eq 6 and compared with the same quantities obtained by ab initio calculations at the CCSD(T)(F12c)/cc-pVDZ-F12 level after correction for basis set superposition error. Equation 6 involves only properties of the isolated molecules B and HX, namely, the minimum value σ_min_(B) of the MESP on the 0.001 e/bohr^3^ iso-surface of B, the maximum value σ_max_(HX) of the MESP on the 0.001 e/bohr^3^ iso-surface of HX, the reduced nucleophilicity И_B_ of B, and the reduced electrophilicity Ξ_HX_ of HX. The molecules HX are all linear and therefore in each case σ_max_(HX) lies on the molecular axis near the H atom. The molecules B were all chosen so that σ_min_(B) can be unambiguously associated with the direction of the axis of a nonbonding electron pair (as conventionally envisaged) and carried by the atom acting as the hydrogen-bond acceptor. И_B_ has recently been identified as an intrinsic property of the atom acting as the hydrogen-bond acceptor, while the reduced electrophilicity Ξ_HX_ is an intrinsic property of the H atom in HX when X is a halogen atom. When X = CN, CCH, or CP, Ξ_HX_ has also a constant value but is different from that of the hydrogen halides. In view of these properties, the comparison of *D*_e_ calculated with eq 6 with the ab initio value of *D*_e_ was made in groups, in each of which the atom acting as the H-bond acceptor was the same, namely, O···HX, followed by N···HX, then C···HX and finally Boron···HX.

The graph of *D*_e_ (eq 6) vs *D*_e_ (ab initio) for each group allowed a linear regression fit with a gradient near to 1, indicating that eq 6 does provide a reasonably accurate method for predicting *D*_e_ from properties of the individual molecules forming the complex. It was noticed, however, that complexes in which HCN was either the H-bond donor or the H-bond acceptor molecule sometimes fell further from the fitted regression line than other complexes. When these were removed from the graph, the scatter was reduced. The reason for this behavior of HCN is not presently known.

## Supporting Information Available

*D*_e_ values calculated from eq 6; graphs of *D*_e_ calculated by eq 6 versus values of *D*_e_ calculated ab initio for groups of complexes B···HX, with complexes involving HCN removed (PDF)
