# Supplementary material for: Hydrogen-Bond Dissociation Energies from the Properties of Isolated Monomers
Source: J Phys Chem A. 2023 May 19;127(21):4715–23. doi: 10.1021/acs.jpca.3c02159 (PMC10240493; doi:10.1021/acs.jpca.3c02159)
Supplement: Supplementary file 1 — jp3c02159_si_001.pdf [file jp3c02159_si_001.pdf]

Supporting Information for Publication with

Hydrogen-bond Dissociation Energies from Properties of Isolated Monomers

Ibon Alkorta*,[a] and Anthony Legon*,[b]

[a]*Instituto de Química Médica (IQM-CSIC),*

Juan de la Cierva, 3, E-28006 Madrid, Spain,

E-mail: ibon@iqm.csic.es; Tel: +34 915622900

[b] *School of Chemistry, University of Bristol,*

Cantock's Close, Bristol BS8 1TS, U. K.

E-mail: a.c.legon@bristol.ac.uk; Tel: +44 (0)117 331 7708

Index:

Pg. S2-S3 Table S1. Calc. of D_e of complexes R-N···HX from σ_{min}(RN), σ_{max}(HX), $И_B$ and Ξ_{HX} (X= F, Cl, Br, I, CN, CCH, CP)

Pg. S4-S5 Table S2. Calc. of D_e of complexes R-C···HX from σ_{min}(RC), σ_{max}(HX), $И_B$ and Ξ_{HX} (X= F, Cl, Br, I, CN, CCH, CP)

Pg. S6 Table S3. Calc. of D_e of complexes R-B···HX from σ_{min}(RB), σ_{max}(HX), $И_B$ and Ξ_{HX} (X= F, Cl, Br, I, CN, CCH, CP)

Pg. S7 Table S4. Calc. of D_e of complexes B···HX from σ_{min}(B), σ_{max}(HX), $И_B$ and Ξ_{HX} (X= F, Cl, Br, I, CN, CCH, CP)

Pg.S8-S9 Figures S1, S2 and S3

Table S1. Calc. of D_e of complexes R-N···HX from σ_{min}(RN), σ_{max}(HX), $И_B$ and Ξ_{HX} (X= F, Cl, Br, I, CN, CCH, CP)

Lewis base B	σ_{min}(B) (kJ mol^{-1})	σ_{max}(HX) (kJ mol^{-1})	$И_B$	Ξ_{HX}	D_e(calc) (kJ mol^{-1})	D_e(obs) (kJ mol^{-1})	D_e(obs)-D_e(calc)
1. HX = HF							
CH_3CN	-159.2	287.9	0.0333	0.0239	36.5	36.9	0.4
HCN	-133.7	287.9	0.0333	0.0239	30.6	30.3	-0.3
FCN	-119.1	287.9	0.0333	0.0239	27.3	27.4	0.1
N_2	-38.5	287.9	0.0374	0.0239	9.9	9.3	-0.6
PN	-131.5	287.9	0.0374	0.0239	33.8	34.8	0.9
2. HX = HCl							
CH_3CN	-159.2	190.2	0.0333	0.0239	24.1	22.9	-1.2
HCN	-133.7	190.2	0.0333	0.0239	20.2	15.7	-4.5
FCN	-119.1	190.2	0.0333	0.0239	18.0	14.9	-3.1
N_2	-38.5	190.2	0.0374	0.0239	6.5	4.4	-2.1
PN	-131.5	190.2	0.0374	0.0239	22.4	21.8	-0.6
3. HX = HBr							
CH_3CN	-159.2	160.1	0.0333	0.0239	20.3	19.5	-0.8
HCN	-133.7	160.1	0.0333	0.0239	17.0	15.3	-1.7
FCN	-119.1	160.1	0.0333	0.0239	15.2	14.3	-0.9
N_2	-38.5	160.1	0.0374	0.0239	5.5	4.2	-1.3
PN	-131.5	160.1	0.0374	0.0239	18.8	18.5	-0.3
4. HX = HI							
CH_3CN	-159.2	119.4	0.0333	0.0239	15.1	14.2	-0.9
HCN	-133.7	119.4	0.0333	0.0239	12.7	10.9	-1.8
FCN	-119.1	119.4	0.0333	0.0239	11.3	10.4	-0.9
N_2	-38.5	119.4	0.0374	0.0239	4.1	3.1	-1.0
PN	-131.5	119.4	0.0374	0.0239	14.0	13.4	-0.6
5. HX =HCN							
CH_3CN	-159.2	216.9	0.0333	0.0164	18.9	24.2	5.3
HCN	-133.7	216.9	0.0333	0.0164	15.8	15.3	-0.5
FCN	-119.1	216.9	0.0333	0.0164	14.1	17.5	3.4
N_2	-38.5	216.9	0.0374	0.0164	5.1	5.0	-0.1
NP	-131.5	216.9	0.0374	0.0164	17.5	21.9	4.4
6. HX = HCCH							
CH_3CN	-159.2	134.8	0.0333	0.0164	11.7	12.5	0.8
HCN	-133.7	134.8	0.0333	0.0164	9.8	10.1	0.3
FCN	-119.1	134.8	0.0333	0.0164	8.8	9.3	0.5
N_2	-38.5	134.8	0.0374	0.0164	3.2	3.1	-0.1

PN	-131.5	134.8	0.0374	0.0164	10.9	11.5	0.6
7. HX =HCP							
CH_3CN	-159.2	126.3	0.0333	0.0164	11.0	12.4	1.4
HCN	-133.7	126.3	0.0333	0.0164	9.2	9.9	0.7
FCN	-119.1	126.3	0.0333	0.0164	8.2	9.1	0.9
N_2	-38.5	126.3	0.0374	0.0164	3.0	3.0	0.0
PN	-131.5	126.3	0.0374	0.0164	10.2	11.5	1.3

Table S2. Calc. of D_e of complexes R-C···HX from σ_{min}(RC), σ_{max}(HX), $И_B$ and Ξ_{HX} (X= F, Cl, Br, I, CN, CCH, CP)

Lewis base B	σ_{min}(B) (kJ mol^{-1})	σ_{max}(HX) (kJ mol^{-1})	$И_B$	Ξ_{HX}	D_e(calc) (kJ mol^{-1})	D_e(obs) (kJ mol^{-1})	D_e(obs)-D_e(calc)
HX = HF							
SC	-119.7	287.9	0.0349	0.0239	28.7	29.0	0.3
OC	-58.5	287.9	0.0349	0.0239	14.0	14.2	0.2
HNC	-138.9	287.9	0.0337	0.0239	32.2	31.9	-0.3
FNC	-106.9	287.9	0.0337	0.0239	24.8	24.4	-0.4
CH_3NC	-161.8	287.9	0.0337	0.0239	37.5	37.6	0.1
HX =HCl							
SC	-119.7	190.2	0.0349	0.0239	19.0	13.5	-5.5
OC	-58.5	190.2	0.0349	0.0239	9.3	14.9	5.6
FNC	-106.9	190.2	0.0337	0.0239	16.4	12.6	-3.8
HNC	-138.9	190.2	0.0337	0.0239	21.3	19.4	-1.9
CH_3NC	-161.8	190.2	0.0337	0.0239	24.8	23.6	-1.2
HX = HBr							
SC	-119.7	160.1	0.0349	0.0239	16.0	15.0	-1.0
OC	-58.5	160.1	0.0349	0.0239	7.8	6.3	-1.5
FNC	-106.9	160.1	0.0337	0.0239	13.8	12.2	-1.6
HNC	-138.9	160.1	0.0337	0.0239	17.9	16.3	-1.6
CH_3NC	-161.8	160.1	0.0337	0.0239	20.9	20.3	-0.6
HX = HI							
SC	-119.7	119.4	0.0349	0.0239	11.9	10.6	-1.3
OC	-58.5	119.4	0.0349	0.0239	5.8	4.4	-1.4
FNC	-106.9	119.4	0.0337	0.0239	10.3	8.6	-1.7
HNC	-138.9	119.4	0.0337	0.0239	13.4	11.5	-1.9
CH_3NC	-161.8	119.4	0.0337	0.0239	15.6	14.5	-1.1
HX =HCN							
SC	-119.7	216.9	0.0349	0.0164	14.9	16.5	1.6
OC	-58.5	216.9	0.0349	0.0164	7.3	7.1	-0.2
FNC	-106.9	216.9	0.0337	0.0164	12.8	14.3	1.5
HNC	-138.9	216.9	0.0337	0.0164	16.7	19.0	2.3
CH_3NC	-161.8	216.9	0.0337	0.0164	19.4	23.1	3.7
HX =HCCH							
SC	-119.7	134.8	0.0349	0.0164	9.2	8.8	-0.4
OC	-58.5	134.8	0.0349	0.0164	4.5	4.2	-0.3
FNC	-106.9	134.8	0.0337	0.0164	8.0	7.6	-0.4
HNC	-138.9	134.8	0.0337	0.0164	10.3	9.9	-0.4

CH_3NC	-161.8	134.8	0.0337	0.0164	12.1	11.8	-0.3
HX =HCP							
SC	-119.7	126.3	0.0349	0.0164	8.7	8.8	0.1
OC	-58.5	126.3	0.0349	0.0164	4.2	4.1	-0.1
FNC	-106.9	126.3	0.0337	0.0164	7.5	7.5	0.0
HNC	-138.9	126.3	0.0337	0.0164	9.7	9.7	0.0
CH_3NC	-161.8	126.3	0.0337	0.0164	11.3	11.8	0.5

Table S3. Calc. of D_e of complexes R-B···HX from σ_{min}(RB), σ_{max}(HX), $И_B$ and Ξ_{HX} (X= F, Cl, Br, I, CN, CCH, CP)

Lewis base B	σ_{min}(B) (kJ mol^{-1})	σ_{max}(HX) (kJ mol^{-1})	$И_B$	Ξ_{HX}	D_e(calc) (kJ mol^{-1})	D_e(obs) (kJ mol^{-1})	D_e(obs)-D_e(calc)
1. HX = HF							
H_3C-B	-160.3	287.9	0.0368	0.0239	40.6	40.5	-0.1
H-B	-134.5	287.9	0.0368	0.0239	34.1	33.4	-0.7
F-B	-89.3	287.9	0.0368	0.0239	22.6	21.5	1.1
2. HX = HCl							
H_3C-B	-160.3	190.2	0.0368	0.0239	26.8	26.3	-0.5
H-B	-134.5	190.2	0.0368	0.0239	22.5	20.5	-2.0
F-B	-89.3	190.2	0.0368	0.0239	14.9	12.8	-2.1
3. HX = HBr							
H_3C-B	-160.3	160.1	0.0368	0.0239	22.6	23.8	1.2
H-B	-134.5	160.1	0.0368	0.0239	18.9	18.0	-0.9
F-B	-89.3	160.1	0.0368	0.0239	12.6	10.9	-1.7
4. HX = HI							
H_3C-B	-160.3	119.4	0.0368	0.0239	16.8	16.7	-0.1
H-B	-134.5	119.4	0.0368	0.0239	14.1	12.6	-1.5
F-B	-89.3	119.4	0.0368	0.0239	9.4	7.7	-1.7
5. HX = HCN							
H_3C-B	-160.3	216.9	0.0368	0.0164	21.0	22.3	1.3
H-B	-134.5	216.9	0.0368	0.0164	17.6	17.3	-0.3
F-B	89.3	216.9	0.0368	0.0164	11.7	11.4	-0.3
6. HX = HCCH							
H_3C-B	-160.3	134.8	0.0368	0.0164	13.0	11.3	-1.7
H-B	-134.5	134.8	0.0368	0.0164	10.9	9	-1.9
F-B	-89.3	134.8	0.0368	0.0164	7.3	6.1	-1.2
7. HX = HCP							
H_3C-B	-160.3	126.3	0.0368	0.0164	12.2	11.4	-0.8
H-B	-134.5	126.3	0.0368	0.0164	10.3	8.9	-1.4
F-B	-89.3	126.3	0.0368	0.0164	6.8	6.1	-0.7

Table S4. Calc. of D_e of complexes B···HX from σ_{min}(B), σ_{max}(HX), $И_B$ and Ξ_{HX} (X= F, Cl, Br, I, CN, CCH, CP)

CO···HX	σ_{min}(B) (kJ mol^{-1})	σ_{max}(HX) (kJ mol^{-1})	$И_B$	Ξ_{HX}	D_e(calc) (kJ mol^{-1})	D_e(obs) (kJ mol^{-1})	D_e(obs)-D_e(calc)
CO···HF	-25.0	287.9	0.0376	0.0239	6.5	6.9	0.4
CO···HCl	-25.0	190.2	0.0376	0.0239	4.3	4.1	-0.2
CO···HBr	-25.0	160.1	0.0376	0.0239	3.6	3.4	-0.2
CO···HI	-25.0	119.4	0.0376	0.0239	2.7	2.6	-0.1
CO···HCN	-25.0	216.9	0.0376	0.0164	3.3	4.2	0.9
CO···HCCH	-25.0	134.8	0.0376	0.0164	2.1	2.7	0.6
CO···HCP	-25.0	126.3	0.0376	0.0164	1.9	2.6	0.7
ClCN···HX							
ClCN···HF	-127.5	287.9	0.0333	0.0239	29.2	29.7	0.4
ClCN···HCl	-127.5	190.2	0.0333	0.0239	19.3	18.6	-0.7
ClCN···HBr	-127.5	160.1	0.0333	0.0239	16.2	15.6	-0.7
ClCN···HI	-127.5	119.4	0.0333	0.0239	12.1	10.9	-1.2
ClCN···HCN	-127.5	216.9	0.0333	0.0164	15.1	19.3	4.2
ClCN···HCCH	-127.5	134.8	0.0333	0.0164	9.4	10.3	0.9
ClCN···HCP	-127.5	126.3	0.0333	0.0164	8.8	10.1	1.3
ClNC···HX							
ClNC···HF	-124.0	287.9	0.0337	0.0239	28.8	28.5	-0.3
ClNC···HCl	-124.0	190.2	0.0337	0.0239	19.0	17.3	-1.7
ClNC···HBr	-124.0	160.1	0.0337	0.0239	16.0	14.7	-1.3
ClNC···HI	-124.0	119.4	0.0337	0.0239	11.9	17.0	5.1
ClNC···HCN	-124.0	216.9	0.0337	0.0164	14.9	11.0	-3.9
ClNC···HCCH	-124.0	134.8	0.0337	0.0164	9.2	8.9	-0.3
ClNC···HCP	-124.0	126.3	0.0337	0.0164	8.7	8.8	0.2

Figures S1, S2 and S3. Graphs of D_e(eq.6) versus D_e(ab initio) for hydrogen-bonded complexes B···HX

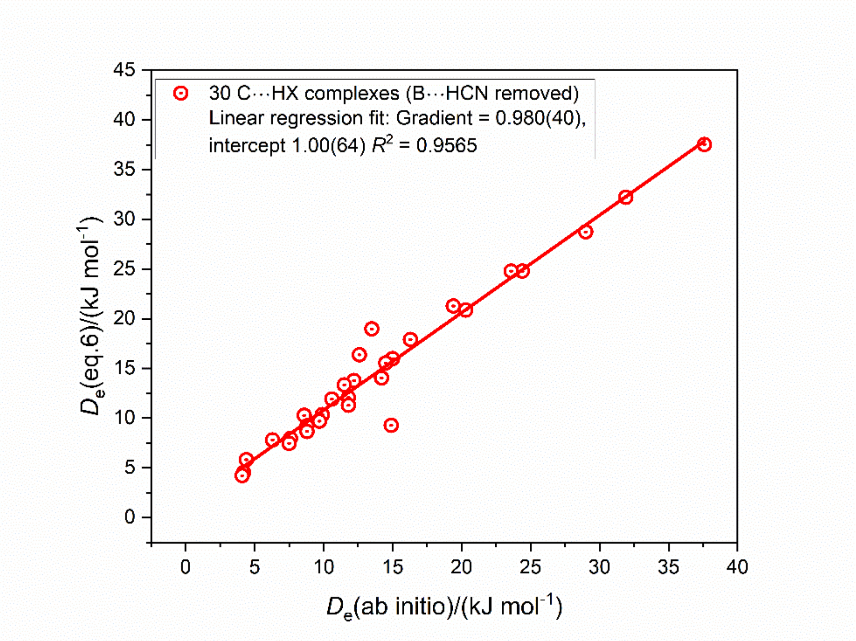

Figure S1. Comparison of D_e of B···HX calculated from eq.(6) with those obtained by ab initio calculations for 30 complexes containing C···HX hydrogen bonds after the five complexes involving HCN are removed from Figure 5

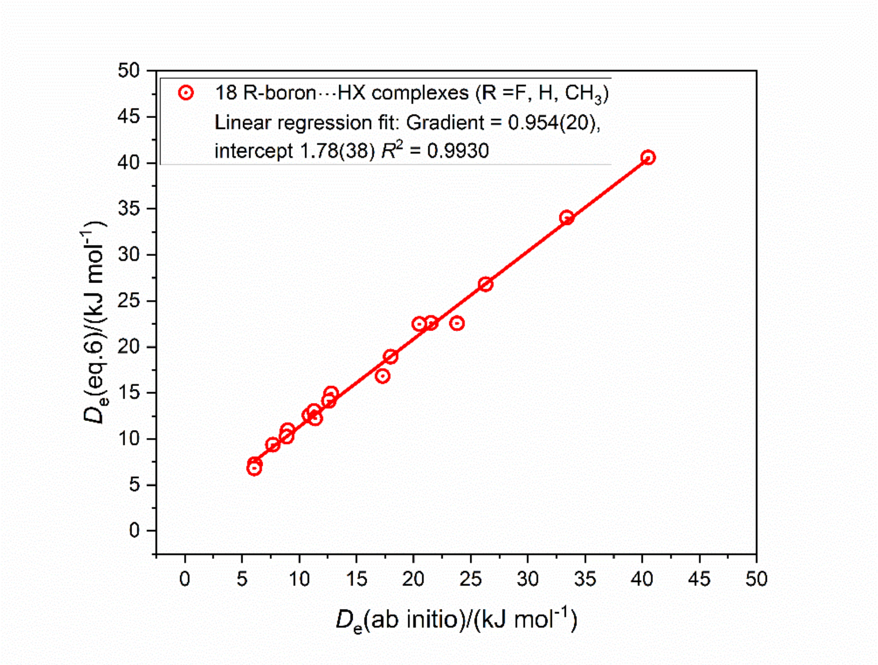

Figure S2. Comparison of D_e of B···HX calculated from eq.(6) with those obtained by ab initio calculations for 18 complexes containing R-boron···HX hydrogen bonds after the three complexes involving HX = HCN are removed from Figure 6.

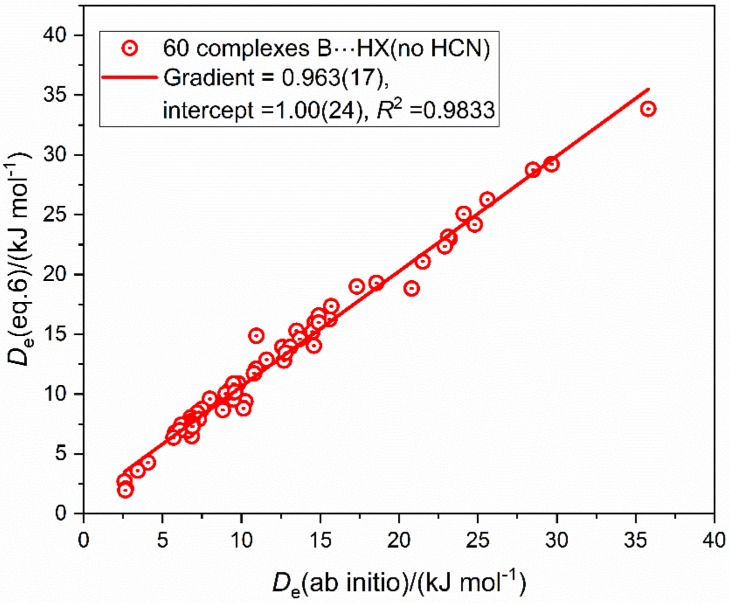

Figure S3. D_e(eq.6) versus D_e(ab initio) for 60 complexes B···HX. The complexes involve the Lewis bases CO, ClCN, ClNC and R-B (where R = H_3Si, Cl , Br, I , CN, NC and F_3C) and were chosen because they were not involved in the determination of the reduced nucleophilicities $И_B$ of the Lewis bases or the reduced electrophilicities Ξ_{HX} of the Lewis acids. This figure corresponds to that in Figure 7, except that 10 complexes involving HCN have been removed.
